# Genomic Resources for *Salminus brasiliensis*

**DOI:** 10.3389/fgene.2022.855718

**Published:** 2022-03-28

**Authors:** Raissa Cristina Dias Graciano, Rafael Sachetto Oliveira, Isllas Miguel Santos, Gabriel M. Yazbeck

**Affiliations:** ^1^ Laboratório de Recursos Genéticos, Programa de Pós Graduação Em Biotecnologia, Universidade Federal de São João Del Rei, São João Del Rei, Brazil; ^2^ Departamento de Ciência da Computação, Universidade Federal de São João Del Rei, São João Del Rei, Brazil; ^3^ Laboratório de Recursos Genéticos, Departamento de Zootecnia, Universidade Federal de São João Del Rei, São João Del Rei, Brazil

**Keywords:** bioinformatics, sequencing-by-synthesis, population genetics resources, hatchery, environmental management, characiformes

## Abstract

The Neotropical region bears the most diverse freshwater fish fauna on the planet and is the stage for dramatic conservation struggles. Initiatives aiming for conservation of a single emblematic fish, a flagship species, to which different onlookers relate on a cultural/personal level, holds promise towards engagement and conservation actions benefiting whole biological communities and ecosystems. Here, we present the first comprehensive genomic resources for *Salminus brasiliensis*, a potential flagship Neotropical species. This fish faces pressing conservation issues, as well as taxonomic uncertainty, being a main species relevant to angling and commercial fisheries. We make available 178 million Illumina paired-end reads, 90 bases long, comprising 16 Gb (≈15X coverage) of filtered data, obtained from a primary genomic library of 500-bp fragments. We present the first *de novo* genomic assembly for *S. brasiliensis*, with ∼1 Gb (*N*_50_ = 10,889), as well as the coding genome annotation of 12,962 putative genes from assembled genomic fragments over 10 kb, most of which could be identified from the Ostariophysi GenBank database. We also provide a genome-wide panel for more than 80,000 predicted microsatellite loci for low-cost, fast and abundant DNA marker development for this species. A total of 47, among 52 candidates, empirically assayed microsatellites were confirmed as polymorphic in this fish. All genomic data produced for *S. brasiliensis* is hereby made publicly accessible. With the disclosure of these results, we intend to foster general biology studies and to provide tools to be applied immediately in conservation and aquaculture in this candidate flagship Neotropical species.

## Introduction

The Neotropical region bears the most diverse freshwater fish fauna on the planet, apparently as the result of rapid speciation process (Melo et al., 2021). Freshwater systems and fish biodiversity face ever-increasing impacts from human activity, a global issue that often does not muster as much societal awareness (Su et al., 2021) as other pressing conservation challenges such as accelerated rates of deforestation and normative deregulation (Gonçalves et al., 2020; Gonçalves-Souza et al., 2021). Associated with its particularly rich fish fauna, South America harbors some of the largest river basins on Earth and it is the stage for dramatic conservation struggles involving conflicting stakeholder concerns. The degree of cumulative anthropogenic degradation of its aquatic systems is worrisome, as epitomized by two catastrophic mine-waste spills caused by obsolete tailing dam failures in Brazil in 2015 (Fernandes et al., 2016) and 2019 (Vergilio et al., 2020), leading to severe effects upon freshwater biodiversity (e.g., Gomes et al., 2019), in addition to social disruption and loss of human life (Polignano and Lemos, 2020).

Conservation initiatives aiming a single emblematic species, to which large numbers of diverse onlookers can relate on a personal or social level, holds promise towards engagement and successful actions for the benefit of whole biological communities and ecosystems (e.g., Leader-Williams et al., 1990; Dietz et al., 1994). For different taxonomic groups and geographic locations, some more well-known, culturally or economically important species can act as flagship species (e.g., Ochieng et al*.* 2021; Preston et al., 2021). Still, among those characterized as the 20 most charismatic species, none is a fin fish (Albert et al., 2021), and a recent assessment in Brazil noted only one Amazonian teleost fish, *Araipamas gigas*, as a flagship species, among 62 elected taxa (Wosnick et al., 2021). In this regard, the singular Neotropical migratory fish fauna, also known as *piracema* fish (Carolsfeld et al., 2004), are under-represented, especially given their prominent social-environmental importance, fragile conservation status and general public awareness.

**FIGURE 1:**
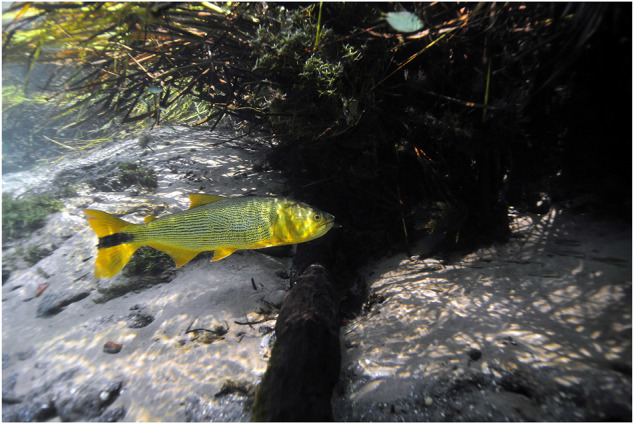
*Salminus brasiliensis* in its natural habitat, in Bonito, MS, Brazil (Author: André Seale).

One particular migratory species arguably fits the flagship bill better than most: *Salminus brasiliensis* Cuvier 1816 (Characiformes, Bryconidae), also known as *dourado*, *dorado*, *pirayú*, *saipé*, *pirajuba*, *dama* and *picudo* among other common names throughout South America (Froese and Pauley, 2000). It is an emblematic, large (females may reach as much as 26 kg), long-distance swimmer, top-predator with formidable aesthetic appeal (Figure 1). It also enjoys wide recognition from the general public and has established importance in angling, artisanal and commercial fisheries (Feitosa et al., 2004; Gagne et al., 2017). It commonly occurs in the La Plata River Basin, a vast hydrographic system which spans the Paraná, Paraguay and Uruguay Rivers and also in the Patos Lagoon in southern Brazil, being native to a total of five countries, including Argentina, Bolivia, Paraguay and Uruguay. It also has been targeted for aquaculture due to its favorable growth rate, size and gastronomic quality (Crescêncio et al., 2005; Zaniboni-Filho and Schulz, 2003; Zaniboni-Filho et al*.* 2017), despite being a voracious piscivorous species, which could pose challenges to commercial cultivation. *Salminus brasiliensis* is spawned mainly for stock supplementation purposes, along efforts to mitigate the environmental impact of hydroelectric dams upon migratory fish. It has suffered a sharp reduction of natural stocks also because of removal of the riparian zone along rivers, land and water pollution, introduction of invasive species and overfishing. The species has not yet been included, however, in the Red Book of Threatened Species (Rosa and Lima, 2008), mainly given its relatively high abundance in the Pantanal region of the Paraguay Basin. Yet, it has been considered vulnerable in the Paraná Basin (Marques et al., 2002; Abilhoa, 2004) and highly vulnerable in the La Plata River (Zayas and Cordiviola, 2007). It also has been classified as virtually extinct, with population size below a viable threshold, in some major Paraná Basin locations, such as the Grande, Tietê and Paranapanema Rivers (Rosa and Lima, 2008).

Rosso et al. (2012) observed the differentiation among *S. brasiliensis* stocks from Argentina’s Pampa Plain and other parts of South America. According to Machado et al. (2017), the species displays distinct haplogroups, with the Upper Paraná containing a distinct Evolutionary Significant Unit from the remaining La Plata River Basin. Thus, there are taxonomic uncertainties associated with this species and further work is needed to inform its conservation and management plans.

Genetic and genomic resources can be applied to resolve conservation and taxonomic issues. So far, eight polymorphic microsatellites were described for this species by Rueda et al. (2011) and another 47 by our own group (*in* Cao et al., 2016) from next-generation sequencing (NGS) data first fully disclosed herein. Brandão-Dias et al. (2014) provided the first cytoplasmic genomic resources for *S. brasiliensis* with publication of its assembled and annotated mitogenome (17,721 bp long). Here, we present the first comprehensive genomic resources for *Salminus brasiliensis*, contributing to basic molecular genetics knowledge, and to resolution of conservation and taxonomic challenges.

## Materials and Methods

### DNA Extraction and Sequencing

The genomic DNA sample from *S. brasiliensis* was obtained from a single female specimen from the Itutinga Hatchery at Grande River, in the Upper Paraná Basin, located in Minas Gerais–Brazil (21°17′040″S, 44°37′023″W). Following euthanasia, the fish was held on in ice during transport to the laboratory, where a muscle tissue sample was removed and placed in liquid nitrogen until DNA extraction. The individual was kept as a voucher (LARGE130547^1^). All procedures were authorized by UFSJ’s Ethics Committee on Animal Research (CEUA-UFSJ); specimen collection was conducted under SISBIO license number 37222 and genetic patrimony access was granted through license CGEN - A9D0E51. No experimentation on live animals was conducted in this research. DNA extraction, library preparation and NGS steps were conducted as presented in Yazbeck et al. (2018). In summary, a single primary library of random genomic fragments (∼500 bp) was sequenced using an Illumina HiSeq 2000, generating 90-bp paired-end short reads. Raw data was filtered for quality and removal of duplicates and stored as two corresponding FASTQ files.

### Bioinformatics

We verified FASTQ quality values with FastQC (ver. 0.11.09–Andrews, 2010) and we used KmerGenie (Chikhi and Medvedev, 2014) to assess *k* values from 41 to 61, in order to search which *k*-mer would result in a more diverse dataset. Then, different preliminary *de novo* genomic assemblies were attempted from the generated short reads, assaying best *k*-mer values with Minia (Chikhi and Rizk, 2012). The chosen *k*-mer was used for a *de novo* assembly using SOAPdenovo 2 (Luo et al*.*, 2012). Assembly qualities were inferred by search for single-copy conserved core eukaryotic ortholog genes with BUSCO (Benchmarking universal Single-Copy Orthologs) (Simão et al*.*, 2015), using the Zebrafish, *Danio rerio*, database (BuscoDB). The procedure to map perfect-repeat microsatellite loci (potential candidates for new molecular markers) in the chosen assembly was also conducted according to the methodology detailed in Yazbeck et al. (2018). In summary, an unknown genomic assembly performed with these short reads was used for potential microsatellite loci mining by a service provider. We used the data produced by this step, namely predicted PCR products, as queries to be searched in the new chosen assembly, using BLAST (Boratyn et al., 2013). The filtered short reads were mapped onto the chosen assembly using the SOAP aligner, and the resulting file was converted into BAM format with the aid of SAMtools (Li et al., 2009).

Based on an estimate of the average eukaryotic gene sequence length of 12,000 bp (Cooper and Hausman, 2016), due to the fragmented nature of the assemblies and computational power restrictions, we proceeded to perform functional annotation of contigs and scaffolds larger than 10,000 bases only, using the MAKER pipeline (Cantarel et al., 2008) using the Characiformes database^2^ (peptide sequences and expressed sequence tags) from NCBI as a training set. Annotated sequences were then characterized by BLAST, from the superorder Ostariophysi data available from NCBI^3^ and functionally classified with the aid of PANTHER (Mi et al., 2010). The mitogenome was obtained according to the steps presented in Yazbeck et al. (2014).

## Results

The library sequencing resulted in a pair of twin FASTQ files consisting of 90-bp paired-end reads amounting to around 16 Gb of filtered data, with 97.4% exhibiting PHRED *Q* quality values of 20 or more (1% or less of wrong base calls). The average quality value per read was *Q* = 38 and CG content was 41.05%. This data is now available at NCBI’s Sequence Read Archive (SRA) (Supplementary Material S1-under accession number PRJNA792751).

Even though *k*-mer analysis indicated *k* = 41 as a best candidate, three different assembly attempts were assayed, with *k* = 41, 47 and 55, using MINIA and SOAPdenovo 2. Table 1 shows the number of complete core eukaryotic genes retrieved from each assembly, with the *k* = 41 assembly performing less well than *k* = 47 (98 and 194 complete genes, respectively). Both attempts performed with SOAPdenovo 2 (*k* = 47 and 55) resulted in 0.92 and 0.96 Gb assemblies (Table 2), with the former presenting a superior *N*_50_ value (10,889). Thus, the *k* = 47 assembly was selected for the annotation process and used as the basis for microsatellite mapping. It consisted of 560,090 contigs or scaffolds varying from 100 to 134135 bases (average 1,643) (Supplementary Material S2). Only 28 scaffolds were larger than 100 kb, 24,106 were larger than 10 kb, and 115,292 larger than 1 kb. This assembly showed a CG content of 41.25%.

**TABLE 1:** Results from the search of a set of 303 core eukaryotic orthologous genes in alternative assemblies performed with different *k*-mer values, according to BUSCO.

Gene	*k* = 41	*k* = 47	*k* = 55
Complete	98	194	58
Complete and single-copy	96	191	57
Complete and duplicated	2	3	1
Fragmented	148	80	113
Missing	57	29	132

**TABLE 2:** Description of the *S. brasiliensis* genomic assemblies performed with SOAPdenovo 2.

*k*-mer size	Genome size (bp- including N*)	Genome size (not including N*)	*N*_50_	Number of scaffolds
*k* = 55	955,747,686	913,685,574	10,567	1,026,499
*k* = 47	920,486,504	874,381,160	10,889	560,090

*N = unidentified base calls.

An undisclosed assembly with these short reads by a service provider, followed by microsatellite search, revealed 86,832 potential microsatellite loci. These loci were searched with BLAST using the predicted PCR products and mapped in our new chosen *k* = 47 assembly, and the results are available in Supplementary Table S1. Around 76% of predicted loci could be found with a 100% match (66,206 microsatellites), another ∼18% (14,527) were found with similarity of 90% or above and ∼0.9% 742) with similarity within the 77–89% range. Around 6% of microsatellite loci determined from the unknown assembly (5,357) could not be mapped back on the assembly we utilized, including 13 loci empirically validated (out of 47) as polymorphic markers (Cao et al., 2016).

**FIGURE 2:**
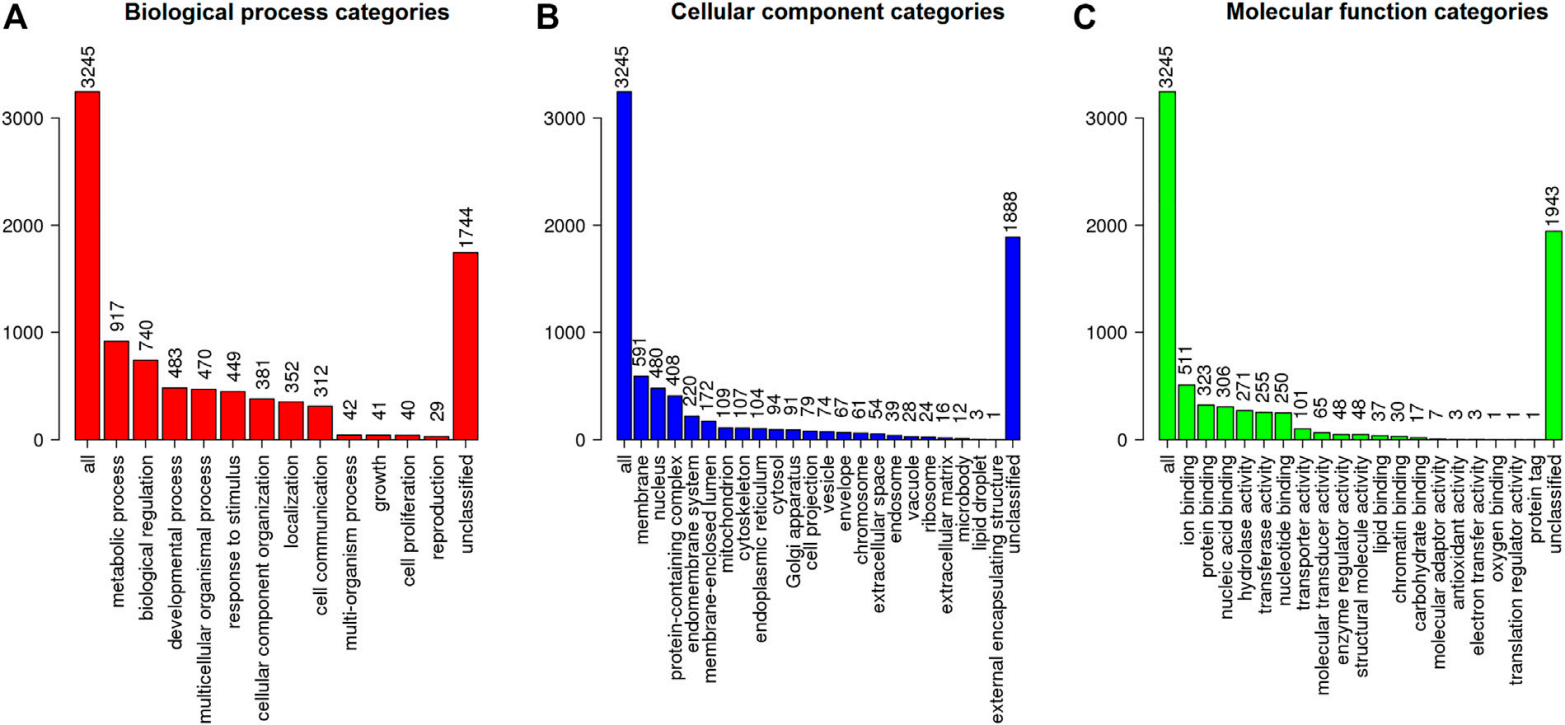
Functional classification (according to Gene Ontology) regarding the **(A)** biological process, **(B)** cellular component, and **(C)** molecular function for predicted genes from *S. brasiliensis*.

The MAKER prediction pipeline resulted in the annotation of 12,962 genes from around 485 Mb of analysed sequences from *S. brasiliensis,* with gene, exon and intron average sizes of 6,125, 194 and 760 bp, respectively. These data were consolidated as a general feature format file (GFF3), with detailed description of coding sequences (CDS), hereby made available through FigShare (Supplementary Material S3). The genes found then were searched through BLAST against the Ostariophysi databank from NCBI, allowing the characterization of 10,211 genes (Supplementary Table S2), which were functionally classified according to Gene Ontology regarding their potential biological role, resulting in the elucidation of function of 3,245 *S. brasiliensis* genes (Figure 2).

The sequenced individual’s mitogenome is deposited in GenBank under accession KY825190 and amounts to 17,998 bp.

## Discussion

Genomics can produce powerful tools for fish conservation (Bernos et al., 2020), and we here provide the first comprehensive genomic data on *S. brasiliensis*. This study generated a massive set of short DNA reads with associated base-call quality scores, which is now publicly available at NCBI’s Sequence Read Archive, allowing its application by other research groups. This data set, even though of low genomic coverage, is considered of high quality (average of one error per each 6,250 bp). It allowed the exploration of different *de novo* genomic assemblies, which showed that the adoption of different *k*-mer parameters resolve different genomic regions, as judged by complete core eukaryotic genes analysis. We make available one assembly (*k* = 47), while very fragmented, as shown by its *N*_50_ and short contigs/scaffolds, it still allowed the annotation of over 12,000 protein-encoding sequences, whereas only 13 mitochondrial genes were previously available (Brandão-Dias et al., 2014). Among arbitrary examples of fully annotated genes from this assembly is the *Leucine rich repeat containing 10* gene (LRRC10) involved in heart-tissue regeneration in *Astyanax mexicanus* (Stockdale et al., 2018) and a gonadotrophin-releasing hormone (GnRH) gene, which could support biotechnological development for induced hatchery spawning of this species, as it is involved in gonadal maturation. This functional annotation is made available as a GFF3 file. The number of annotated genes found (almost 13,000) leads us to believe that the presented assembly resolves approximately 40–50% of this species’ genome, judging from comparison with completely characterized genomes from other fishes (*e.g.* 30,741 coding sequences from *Danio rerio* - Howe et al*.,* 2013).

We also provided a panel of tens of thousands of candidate microsatellite loci, which can serve as a starting point for fast and inexpensive molecular marker development in this species, allowing development of marker loci for stock delimitation, linkage mapping, QTL characterization and association studies. Previously, Cao et al. (2016) validated 47 polymorphic microsatellite loci from this panel, attesting to its utility for efficient molecular marker development. Some microsatellite loci determined from a genomic assembly produced with the short reads presented here, which however could not be traced back to the *k* = 47 assembly, were among these empirically validated markers. We thus vouch for the retention of the non-mapped loci, since it has been shown to produce practical results. There is inherent difficulty in resolving repetitive regions with short reads, which explains the varying results from assembly to assembly. Furthermore, among the more than 5,000 loci not found, it can be shown with other alignment tools (*e.g.* Swipe–Rognes, 2011), that many microsatellite loci are indeed present in different repetition resolutions, searching and matching primer pairs (results not shown), since BLAST has limited power working with low-complexity sequences, such as those present in microsatellite motifs.

The mitogenome of the sequenced specimen showed a larger size (277 bp) than the one previously published (Brandão-Dias et al., 2014), with extra DNA at the D-loop region, allowing rapid identification of putative variable sites to be explored in population genetics and other studies.

Together, the results presented here have the potential to be applied in conservation initiatives and taxonomic investigation of this important large fish, a candidate Neotropical migratory flagship species, aiding resolution of ongoing scientific and environmental issues faced by this organism. It will certainly aid future telomere-to-telomere genome characterization of *S. brasiliensis*.

## Data Availability

The datasets presented in this study can be found in online repositories. The names of the repository/repositories and accession number(s) can be found below: https://www.ncbi.nlm.nih.gov/sra/, PRJNA792751, SRR17407720; https://www.ncbi.nlm.nih.gov/sra/, SRR17486808; https://figshare.com/, 10.6084/m9.figshare.18131495; https://figshare.com/, 10.6084/m9.figshare.11796468; https://figshare.com/, 10.6084/m9.figshare.17754188; https://figshare.com/, 10.6084/m9.figshare.19169756.

## Footnotes

1
https://www.ufsj.edu.br/large.

2
https://www.ncbi.nlm.nih.gov/protein/?term=txid7991%5bOrganism:exp%5d.

3
https:// www.ncbi.nlm.nih.gov/nuccore/?term=txid32519[Organism:exp].
